# Optimization of laser dosimetry based on patient-specific anatomical models for the ablation of pancreatic ductal adenocarcinoma tumor

**DOI:** 10.1038/s41598-023-37859-7

**Published:** 2023-07-08

**Authors:** Pouya Namakshenas, Francesco Maria Di Matteo, Leonardo Bianchi, Eliodoro Faiella, Serena Stigliano, Giuseppe Quero, Paola Saccomandi

**Affiliations:** 1 Department of Mechanical Engineering, Politecnico di Milano, 20156 Milan, Italy; 2 Operative Endoscopy Department, Fondazione Policlinico Universitario Campus Biomedico, Rome, Italy; 3 Radiology Unit, Fondazione Policlinico Universitario Campus Biomedico, Rome, Italy; 4 Pancreatic Surgery Unit, Gemelli Pancreatic Advanced Research Center (CRMPG), Fondazione Policlinico Universitario Agostino Gemelli IRCCS di Roma, Rome, Italy; 5 Università Cattolica del Sacro Cuore di Roma, 00168 Rome, Italy

**Keywords:** Biomedical engineering, Mechanical engineering, Targeted therapies

## Abstract

Laser-induced thermotherapy has shown promising potential for the treatment of unresectable primary pancreatic ductal adenocarcinoma tumors. Nevertheless, heterogeneous tumor environment and complex thermal interaction phenomena that are established under hyperthermic conditions can lead to under/over estimation of laser thermotherapy efficacy. Using numerical modeling, this paper presents an optimized laser setting for Nd:YAG laser delivered by a bare optical fiber (300 µm in diameter) at 1064 nm working in continuous mode within a power range of 2–10 W. For the thermal analysis, patient-specific 3D models were used, consisting of tumors in different portions of the pancreas. The optimized laser power and time for ablating the tumor completely and producing thermal toxic effects on the possible residual tumor cells beyond the tumor margins were found to be 5 W for 550 s, 7 W for 550 s, and 8 W for 550 s for the pancreatic tail, body, and head tumors, respectively. Based on the results, during the laser irradiation at the optimized doses, thermal injury was not evident either in the 15 mm lateral distances from the optical fiber or in the nearby healthy organs. The present computational-based predictions are also in line with the previous ex vivo and in vivo studies, hence, they can assist in the estimation of the therapeutic outcome of laser ablation for pancreatic neoplasms prior to clinical trials.

## Introduction

According to statistics from the American Cancer Society, pancreatic cancer will be the third leading cause of cancer death in the United States by 2022^1,2^. Pancreatic ductal adenocarcinoma (PDAC) is the most common pancreatic neoplasm with a 5-year survival rate of less than 5%^3,4^. Most PDAC tumors (70–80%) are not amenable to surgery and do not respond well to the conventional treatment options like chemotherapy and radiotherapy, in part because they are asymptomatic in the early stages and, thus, diagnosed at advanced phases^5,6^. Laser-induced thermotherapy (LITT) is a minimally invasive technique that induces ablative hyperthermia in tumors through laser-tissue interaction and eradicates the neoplastic cells by high temperatures, above 50−60 °C^7^. Being compatible with endoscopic ultrasound (EUS) and employing the flexible fiber that can be preloaded into a small gauge needle, make the LITT method an ideal choice for the ablation of tumors at hard-to-reach sites^8,9^. Recently, LITT equipped with Nd:YAG laser operating in continuous wave (CW) at 1064 nm has been utilized to treat unresectable PDAC tumors as a part of clinical trials^6^. Although LITT is proven to be viable in ex vivo and in vivo studies, adjustment of laser dosimetry is still challenging for establishing a patient-specific treatment strategy due to the complex tumor microenvironment. Uncontrolled laser irradiance can result in unfavorable damage to the nearby susceptible organs such as major vessels and extensive tissue carbonization, which in turn causes photodegradation of fiber tip^10^.

Numerical modeling has proved to be a powerful tool for developing treatment planning platforms that can assist physicians in predicting and optimizing treatment plans. Di Matteo et al.^11^ investigated the feasibility of numerical simulation in the prediction of ablated volume during the laser photocoagulation of healthy pancreatic tissue under ex vivo conditions by using Nd:YAG laser (CW mode and wavelength of 1064 nm)^11^. Their findings showed a good agreement between the simulation results and ex vivo experimental study on 60 healthy porcine pancreases for the applied laser energy of 1000 J. They found that increasing the laser power from 1.5 to 10 W increases both the intended ablated volume and unwanted carbonization volume to the extent where ablated volume is 25 times greater than the carbonization volume. In the laser power range of 10–20 W, the ablated volume experienced subtle expansion but the carbonization volume increased substantially by 58%, increasing the risk of thermal damage to the adjacent healthy sensitive tissues. Based on their findings, Nd:YAG laser with an output power range between 1.5 and 10 W holds the potential for further research, especially on pancreatic neoplasms. Loiola et al*.*^12^ performed numerical analysis in cylindrical coordinates to predict the hyperthermia thermal damage induced by an Nd:YAG laser beam irradiation, where the volumetric heat source was simulated using the Beer-Lambert law. Their research demonstrated that the model yielded results that were in good agreement with experimental observations from rat liver samples. da Silva et al.^13^ developed parameters of a model for the diode-laser heating of prostate cancer cells in vitro under chemotherapy effects using numerical simulation. Ferreira et al*.*^14^ employed computational modeling to propose an optimal laser ablation treatment strategy for skin cancer, taking into account uncertainties in tissue optical and thermal properties. The study found that uncertainties about tissue properties reported in the literature have little impact on the thermal damage indicator and that the set of mean values of the properties in the model are acceptable. Korganbayev et al*.*^15^ used the advantage of numerical modeling to tune the proportional-integral-derivative (PID) gain parameters for the regulation of LITT in ex vivo porcine pancreases^15^. Prior studies regarding the simulation of LITT in pancreatic tissue involving end-firing Nd:YAG laser were largely concerned with the ex vivo condition, whereas the thermal effects of laser-pancreatic tissue interaction in human in vivo remain poorly understood^11,15,16^. It is important to note that the laser-induced temperature distribution in vivo is heavily influenced by both interfacial convective heat transfer and blood perfusion, where the former arises from blood circulation through main vessels, while the latter occurs when blood pumps into capillary beds. These factors should be taken into consideration in preclinical models^17–19^. In addition to the lack of consideration of the physiological conditions, the prior studies have been conducted without taking into account the individual patient anatomy, which is, indeed, a key aspect of the success of the treatment^20,21^. As preoperative treatment planning systems determine the transient temperature profile and assess tissue damage upon heating in correspondence of the tumor target and surrounding regions, they should be employed as the first step in the thermal ablation process to bring the following assets: decrease the rate of complications, guarantee tumor-free safety margins after ablation, and improve long-term survival.

The literature provides some examples of preoperative thermal and hyperthermal treatment planning systems for liver^22^, cervical^23^, and rectal^24^ tumors, and only one study has been conducted on the ultrasound-based thermal therapy of pancreatic tumors in patient-specific models^25^.

To the best of authors’ knowledge, no previous works have reported the planning of laser ablation for clinical purpose, considering a realistic patient’s anatomy geometry. Indeed, LITT for treating pancreas tumor has started to be investigated only recently, and there are no indications from laser companies about the best settings to be used (as, instead, it happens for all the RFA and MWA devices used in interventional radiology procedures). Moreover, a straightforward monitoring of the thermal effects during the EUS procedure is not available, so the intra-operative control is performed only through the qualitative information given by imaging (i.e. the hyperechoic area progressively surrounded the tip of the fiber^6^). Additionally, there are limited available data on pancreatic tissue in vivo and tumors regarding the ablation volumes for different laser settings (power and time combinations). For all these reasons, having a predictive tool for planning the laser ablation procedure which is applicable to a wide range of power-time combinations is crucial for clinicians. This tool can practically assist physicians in determining the laser dose based on tumor size and location.

Hence, the present work provides a patient-specific LITT model, aiming to adjust the output laser power and irradiation time as the controllable parameters of LITT for optimal pancreatic tumor removal under EUS guidance. Four patients with PDAC tumors in different parts of the pancreas were included in the current study. The patient-specific models were obtained from abdominal magnetic resonance imaging (MRI) segmentation and comprised the pancreas gland, the PDAC tumor, and the surrounding major vessels. A simulation framework developed from the Pennes’ bioheat model was implemented to account for the temperature-dependent thermal and optical properties as well as the changes in the laser deposited energy during the evaporation of water contained in the tissue. The optimization of LITT dosimetry was carried out in CW mode for a wide range of clinical laser powers from 2 to 10 W. To the best of authors’ knowledge, it is the first patient-specific in silico study of LITT for the thermoablation of unresectable PDAC tumors.

## Materials and methods

### Problem definition

**Figure 1:**
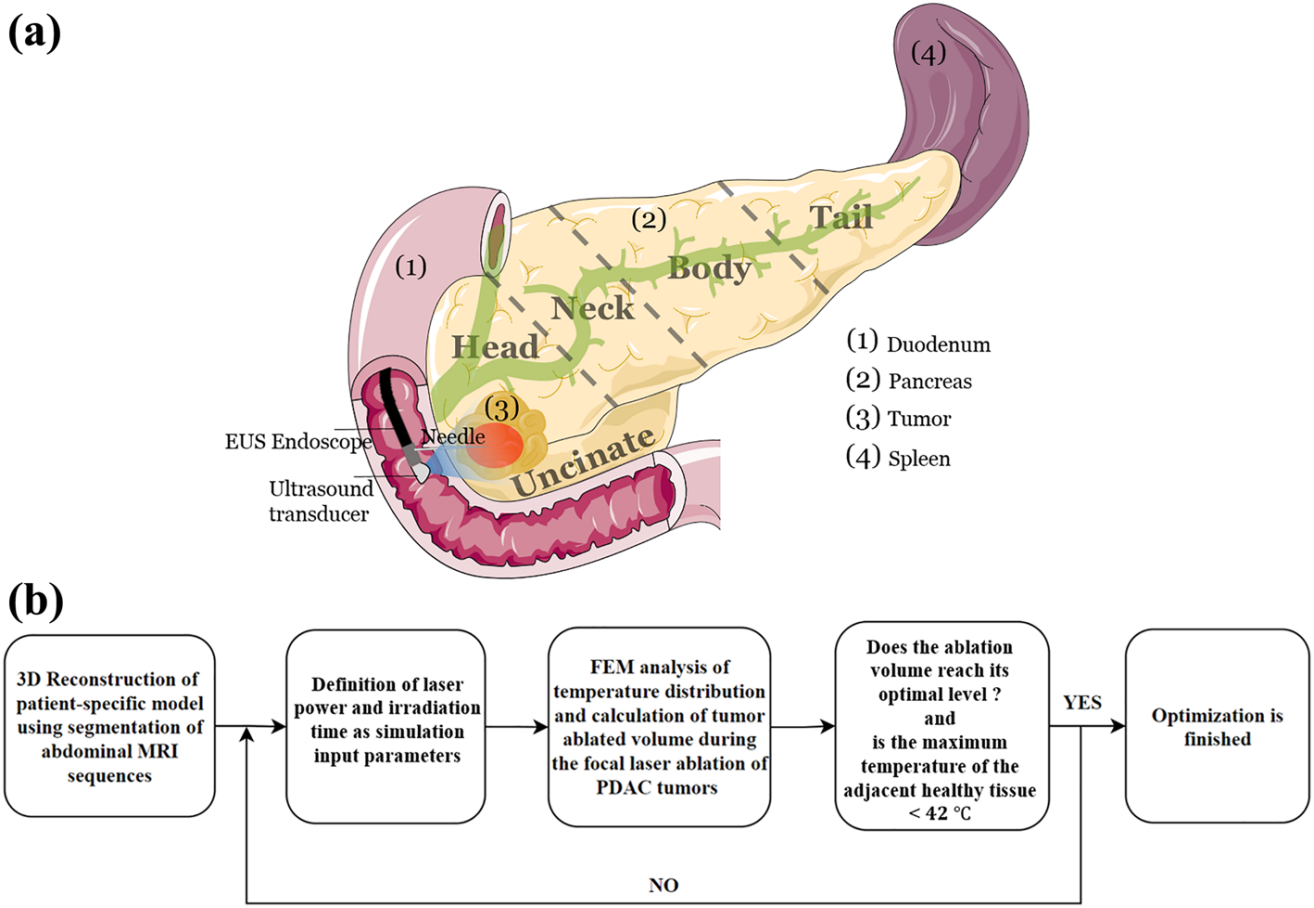
(**a**) Schematic view of the focal laser ablation of pancreatic tumor: Under ultrasound guidance, a laser applicator is inserted into the tumor using a needle and generates the desired amount of heat for tumor coagulation and necrosis through laser irradiation. Parts of the figure were drawn by using pictures from Servier Medical Art. Servier Medical Art by Servier is licensed under a Creative Commons Attribution 3.0 Unported License (https://creativecommons.org/licenses/by/3.0/). (**b**) Steps taken in the present study for the laser dosimetry optimization: the laser power and irradiation time were optimized for each patient model with the goal of removing the tumor volume plus a surrounding safety margin layer at an optimal level while keeping the maximum temperature of the nearby healthy organs below 42 °C.

This study aims to predict the optimal laser power and irradiation time for the ablation of pancreatic neoplasms by developing a mathematical model that takes into account the individual patient’s anatomy. A schematic of the LITT technique for the removal of pancreatic tumor is presented in Fig. 1a. In this technique, the laser applicator is inserted into the tumor tissue to apply the laser energy by a protruded optical fiber (bare fiber, 300 µm diameter) causing the tumor temperature to rise and coagulative necrosis to occur. An Nd:YAG laser with a wavelength of 1064 nm in CW mode was used in the present study, as it has a high penetration depth^26^ and since this wavelength has been used for the first human trial on pancreatic tumors^6^. The pancreas is divided into five main portions, as shown in the anatomy of the pancreas in Fig. 1a: tail, body, neck, head, and uncinate process. Each anatomical location of the pancreas is subjected to a different heat sink effect based on its proximity to major vessels and high blood flow organs. In this simulation, MRI-based anatomical models of patients suffering from PDAC tumors were studied. The laser energy optimization was conducted with the goal of eliminating an optimal percentage of the whole volume of tumor and a safety margin around it while avoiding thermal damage to the major vessels and healthy parenchyma nearby^27^. An optimal ablation percentage would be one in which the whole tumor is treated and hyperthermia is applied to the safety margin around it. The temperature thresholds for determining thermal injury to the sensitive organs and coagulative necrosis of the tumor were considered 42 °C and 60 °C, respectively^18,28–30^. Figure 1b provides the steps taken for the optimization procedure.

### Organ segmentation and 3D geometry construction

The anatomical geometries were derived from the MR images of four patients with PDAC tumors. The study has received approval from the Ethical Committee of the Università Campus Bio-Medico of Roma (86/21 (OSS)) and from the Research Ethical Committee of Politecnico di Milano (Opinion n. 25/2021), thus the study was carried out in accordance with relevant guidelines and regulations. The MR images were anonimized, and informed consent was obtained from the subjects.

**Figure 2:**
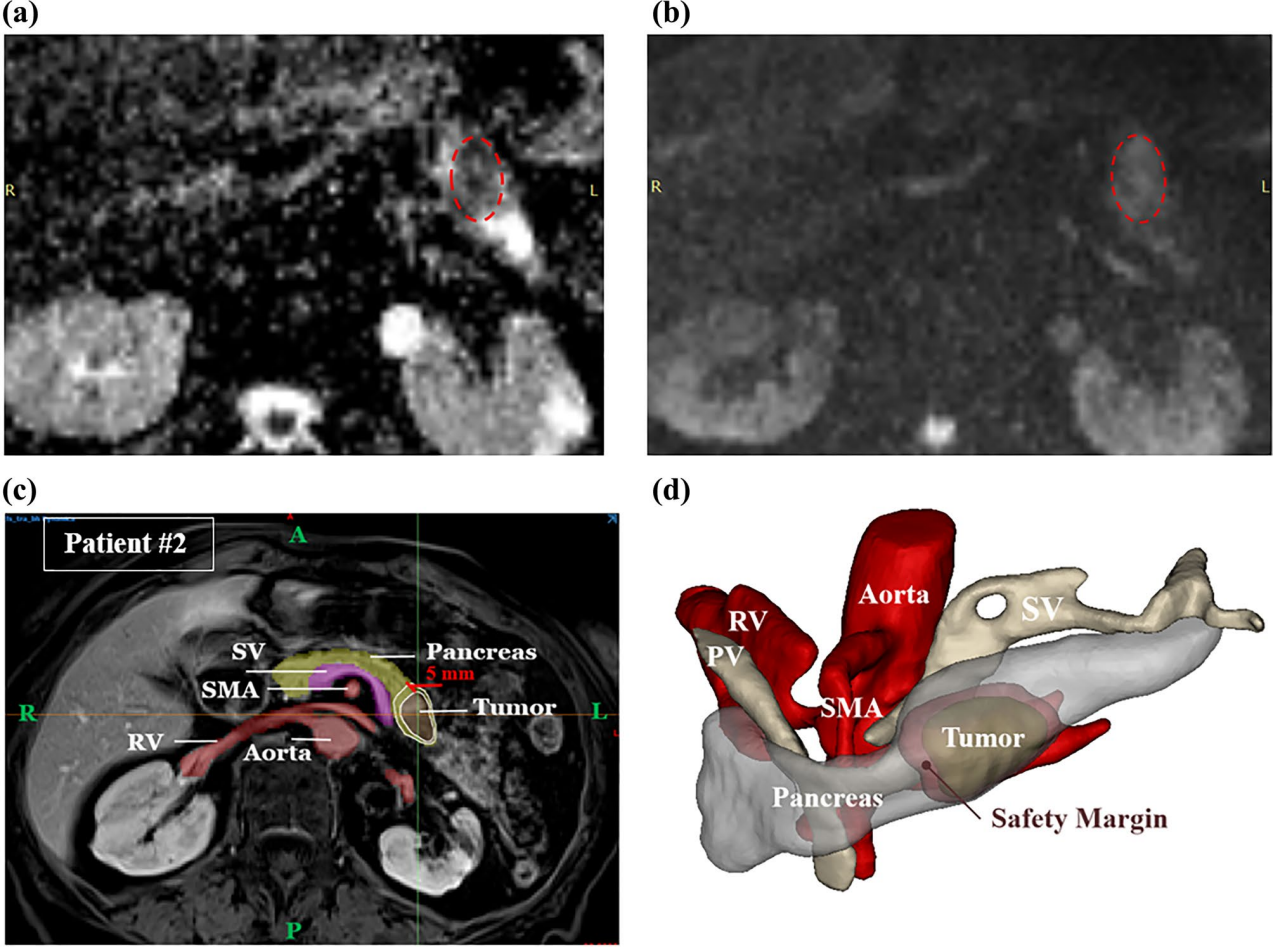
Segmentation of PDAC tumor and adjacent major vessels in abdominal MRI of patient #2; (**a**) apparent diffusion coefficient map, (**b**) diffusion-weighted EPI sequence: the red dashed lines indicate the tumor, (**c**) marked T1-weighted fl3d image: in the body of the pancreas, a solid tumor with an oval morphology and inhomogeneous enhancement caused abrupt interruption of the main pancreatic duct, which shows irregular dilatation downstream along the body-tail, (**d**) reconstructed anatomical geometry. The segmented T1 image was generated using Materialise Mimics® version 21.0, while the anatomical geometry was created using Materialise 3-matic® version 13.0 software (Materialise, Leuven, Belgium, https://www.materialise.com/en). *PV* portal vein, *SV* splenic vein, *SMA* superior mesenteric artery, *RV* renal vein.

Table 1 lists information regarding each patient, as well as the location and volume size of tumors. Materialise Mimics® version 21.0 and Materialise 3-matic® version 13.0 software (Materialise, Leuven, Belgium) were used to perform image processing for organ segmentation and 3D object creation. The obtained 3D object was smoothed and prepared for volume mesh generation, which was then imported as a CAD file for the numerical simulation. Figure 2 shows the key MRI abdominal slice and the anatomical geometry for patient #2. The apparent diffusion coefficient (ADC) map and diffusion-weighted EPI sequence, along with the T1-weighted fl3d images, were used simultaneously to detect the border of PDAC tumors correctly. The geometry includes the pancreas, the tumor, and the major blood vessels surrounding the tumor, as well as the duodenum in the case of the pancreatic head tumor. An artificial layer of pancreatic tissue called safety margin of up to 5 mm was defined around the tumor. In addition to the tumorous region, the safety margin is a treatment target area, ensuring that the laser heat reaches all cancerous cells within the tumor boundary.

**Table 1:** Patients and tumors characteristics.

Patient’s number	Sex	Age	Tumor location	Tumor volume
#1	M	85Y	Tail	3280 $m{m}^{3}$
#2	F	82Y	Body	5560 $m{m}^{3}$
#3	M	73Y	Head	4400 $m{m}^{3}$
#4	F	72Y	Head and uncinate	6740 $m{m}^{3}$

### Mathematical model

In order to predict the tissue thermal behavior during LITT, a modified form of Pennes’ bioheat model was employed:

1 $$\rho C\frac{\partial T}{\partial t}=\nabla \left(k\nabla T\right)-{\rho }_{b}{C}_{b}{\omega }_{b}\left(T-{T}_{b}\right)-{h}_{fg}\frac{dW}{dt}+{Q}_{met}+{Q}_{laser},$$

where $T$ denotes the tissue temperature ($K$), $\rho$ is the density ($kg/{m}^{3}$), $C$ is the specific heat capacity ($J/(kg\cdot K)$), and $k$ is the thermal conductivity ($W/(m\cdot K)$). The second term on the right-hand side of Eq. (1) depicts the heat sink effect due to the blood perfusion during the transport of oxygen and nutrients to the capillary bed from the arteries; where ${\omega }_{b}$ is the blood perfusion rate ($1/s$) and ${T}_{b}$ is the blood temperature that is assumed to be $37 \, ^\circ{\rm C}$. The third term on the right-hand side shows the energy changes associated with the laser-induced evaporation of water within the tumor tissue, where ${h}_{fg}$ is the latent heat of water evaporation ($kJ/kg$), and $W$ is the remaining tissue water content^31^. The fourth and fifth terms on the right-hand side refer to the internal (${Q}_{met}(W/{m}^{3})$) and external (${Q}_{laser}(W/{m}^{3})$) heat generation, respectively, resulting from tissue metabolism and laser irradiation. The laser heat source ${Q}_{laser}$ arises from the absorption of laser photons by the tissue and is described by the Beer-Lambert law as follows^16,32–34^:

2 $${Q}_{laser}={\alpha }_{eff}\cdot I\left(x,y\right)\cdot{e}^{\left(-{\alpha }_{eff}z\right)},$$

where ${\alpha }_{eff}$ (${m}^{-1}$) is a latent variable corresponding to the effective attenuation coefficient that encompasses the laser light absorption and scattering in the irradiated medium (Eq. (3)); $\alpha$ (${m}^{-1}$) is the linear absorption coefficient, ${\alpha }_{s}$ (${m}^{-1}$) is the scattering coefficient, and $g$ is the anisotropy factor.

3 $${\alpha }_{eff}=\sqrt{3\alpha (\alpha +{\alpha }_{s}\left(1-g\right))}.$$

$I\left(x,y\right)$ ($W/{m}^{2}$) is the laser irradiance with a 2D Gaussian profile in every cross-section along the incident path, as described in Eq. (4):

4 $$I\left(x,y\right)={I}_{0}\cdot\mathrm{exp}\left(-\frac{{x}^{2}+{y}^{2}}{2{\sigma }^{2}}\right),$$

where ${I}_{0}$ ($W/{m}^{2}$) is the initial intensity and $\sigma$ ($\mu m$) is the standard deviation. The initial intensity ${I}_{0}$ is proportional to the output laser power ($P (W)$) as defined in Eq. (5). Given that the radius of the bare laser fiber in the present work was ${r}_{f}=150 \mu m$, the standard deviation $\sigma$ was set at $50 \mu m$ (${r}_{f}/3$) to guarantee that 99% of the output laser power goes through the fiber core.

5 $${I}_{0}=\frac{P}{2\pi {\sigma }^{2}}.$$

The thermal damage analysis was performed using a temperature threshold model, as expressed in Eqs. (6) and (7):

6 $$\uptheta \left(\mathrm{t}\right)={\int }_{0}^{{\tau }_{irr}}\frac{1}{{t}_{n}}\left(T>{T}_{d}\right)dt,$$

7 $$\Omega =\mathrm{min}\left(\uptheta ,1\right),$$

where $${\tau }_{irr}$$ is the total time of irradiation during the LITT, $${t}_{n}$$ is the required time for the occurrence of irreversible necrosis if the tissue temperature maintains above the damage temperature ($${T}_{d})$$, $$\uptheta (\mathrm{t})$$ is the indicator of tissue injury, and $$\Omega$$ is the fraction of necrotic tissue. As per Eq. (6), both the irradiation duration and the tissue temperature are contributing to the degree of tissue damage. The ablation volume in the present study is estimated when the fraction of thermal necrosis ($$\Omega$$) is equal to 1^27^.

**Figure 3:**
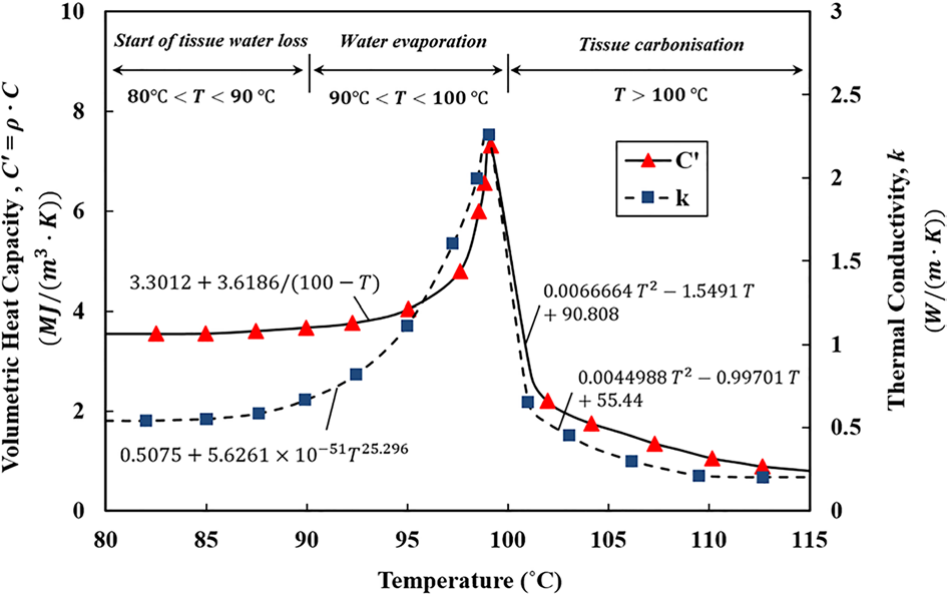
Temperature-dependent values of the volumetric heat capacity ${C}^{^{\prime}}=\rho \cdot C$ and thermal conductivity $k$ which were recorded during ex vivo tests on the bovine liver^37^; the tissue thermal properties increase from $80 ^\circ \mathrm{C}$ up to $100 ^\circ \mathrm{C }$, while this trend reverses as the tissue water evaporation completes at temperatures above $100^\circ \mathrm{C}$.

The present simulation made use of temperature-dependent thermal and optical properties to reflect the realistic response of heat-exposed tissues^35–38^. The tissue water content $W(T)$, as defined in^31^, starts to decrease when the tissue temperature exceeds $80\, ^\circ{\rm C}$, and the evaporation speeds up at around $100\, ^\circ{\rm C}$, resulting in about 50% of tissue water loss at $103\, ^\circ{\rm C}$. Once the temperature goes beyond $104\,\mathrm{^\circ{\rm C} }$, the remaining tissue water content drops exponentially to almost zero, resulting in tissue carbonization^39^. As tissue water evaporates, it transfers energy to low-pressure regions of tissue through a diffusion mechanism and condenses, affecting the tissue thermal properties. Figure 3 illustrates the values of volumetric heat capacity ${C}^{^{\prime}}=\rho \cdot C$ and thermal conductivity $k$ as a function of the tissue temperature. By substituting the dynamic form of volumetric heat capacity ${C}^{^{\prime}}$ in Eq. (1), the water evaporation energy (${h}_{fg}\frac{dW(T)}{dt}$) is implicitly included in the thermal analysis.

The changes in blood perfusion rate as a function of temperature for healthy and tumorous tissue were modeled based on Eqs. (8) and (9), respectively, which were derived from animal models that underwent 30–40 min of heating, with the parameter ${\omega }_{0}$ representing the baseline blood perfusion rate at 37 °C^40,41^. The blood perfusion rate of healthy and tumorous tissue shows a positive peak in the vicinity of 45 °C and 42 °C, respectively, and then decreases as the vasculatures are destroyed by extreme hyperthermia^42^.

8 $${\omega }_{b, healthy}={\omega }_{0}\left\{\begin{array}{c}4.7167\times {10}^{-2} \cdot{T}^{2} - 3.5367 \cdot T + 67.2974, 37\, ^\circ{\rm C} <T\le 42\, ^\circ{\rm C} \\ -{7.7906\times {10}^{-2} \cdot T}^{2} + 9.6329\cdot T - 267.0257, 42\, ^\circ{\rm C} <T\le 45\, ^\circ{\rm C} \\ -15.6972\times {10}^{-2}\cdot {T}^{2}+11.8435 \cdot T- 205.5134, 45 \, ^\circ{\rm C} <T\leq 48\, ^\circ{\rm C} \end{array},\right.$$

9 $${\omega }_{b, tumor}={\omega }_{0}\left\{\begin{array}{c}3.40\times {10}^{-2} \cdot{T}^{2} - 2.5276\cdot T +47.9933, 37\, ^\circ{\rm C} <T\le 42\, ^\circ{\rm C} \\ -0.6891\cdot{ T}^{2} + 58.5436\cdot T - 1241.5792, 42 \, ^\circ{\rm C}<T\leq 44\, ^\circ{\rm C} \end{array}.\right.$$

The thermal properties of native tissues, before exposure to laser irradiation, are given in Table 2. The dynamic thermal properties of tissues are extrapolated from those presented in Fig. 3 to include, in our computational model, the variation of these properties as a function of temperature^37^.

**Table 2:** Tissue thermal properties at normal body temperature (37 °C) used in the present analysis.

Tissue	Density, $\rho$ $kg/{m}^{3}$	Thermal conductivity, $k$ $W/(m\cdot K)$	Specific heat, ${C}_{p}$ $J/(kg\cdot K)$	Blood perfusion rate, ${\omega }_{0}$ $1/s$
Pancreas	1128^59^	0.52^59^	3164^59^	0.018^60^
PDAC Tumor	1128^1^	0.52^1^	3164^1^	0.005^60^
Duodenum	1126^59^	0.53^59^	3690^59^	0.015^25,59^
Soft tissue^2^	1050^25^	0.49^25^	3400^25^	0.003^25,43^

^1^Some parameters of healthy pancreas were used for PDAC tumor.

^2^Soft tissue material was considered between the pancreas and vessels in the model.

The thermal properties used in this study are comparable with the values available in the literature for other tissues^34,43^. Moreover, the uncertainty in tissue properties within the range presented in the literature^14^ has been reported to have a negligible impact on the thermal damage.

According to Table 3, the optical properties of coagulated tissue (${\alpha }_{c}, {\alpha }_{s,c} , {g}_{c})$ differ from those of native tissue (${\alpha }_{n}, {\alpha }_{s,n} , {g}_{n})$. Equations (10), (11) and (12) incorporate the fraction of coagulated tissue $\Omega$ in the calculation of optical properties.

**Table 3:** Optical properties of native and coagulated tissues used for the patient specific model in the present study^38^.

Optical parameter	Native tissue			Coagulated tissue		
	${\alpha }_{n} (1/mm)$	${\alpha }_{s,n} (1/mm)$	${g}_{n}$	${\alpha }_{c} (1/mm)$	${\alpha }_{s,c} (1/mm)$	${g}_{c}$
Value	0.018	4.34	0.93	0.011	30.46	0.92

10 $$\alpha ={\alpha }_{n} \cdot \left(1-\Omega \right)+{\alpha }_{c} \cdot\Omega ,$$

11 $${\alpha }_{s}={\alpha }_{s,n} \cdot \left(1-\Omega \right)+{\alpha }_{s,c} \cdot\Omega ,$$

12 $$g={g}_{n} \cdot \left(1-\Omega \right)+{g}_{c} \cdot\Omega .$$

### Boundary condition and simulation set-up

The anatomical geometry was embedded in a rectangular cube box, with sides positioned 50 mm away from the pancreatic surface. Dirichlet boundary condition of $T=37\, ^\circ{\rm C}$ was applied to the outer surface of the cubic domain in order to avoid an underestimation of thermal damage, as the temperature rise would be forced to slow down if the pancreas surface was to be subjected to the aforementioned temperature boundary condition. The initial temperature of all organs was set to $T=37\, ^\circ{\rm C}$, the normal body temperature. The convective flux boundary condition $h\cdot ({T}_{b}-T)$ was set at the surface of vessels to include circulation heat loss. Here, $h \left(\frac{W}{{m}^{2}\cdot K}\right)$ represents the convective heat transfer coefficient, whose value depends on the caliber of the blood vessels. The convective heat transfer coefficient $h$ for the aorta, superior mesenteric artery, portal vein, splenic vein, and renal vein were considered to be $511 W/({m}^{2}\cdot K)$, $1000 W/({m}^{2}\cdot K)$, $750 W/({m}^{2}\cdot K)$, $750 W/({m}^{2}\cdot K)$, and $750 W/({m}^{2}\cdot K)$, respectively^25^.

The thermal simulation of LITT was conducted in COMSOL Multiphysics software version 5.5 (COMSOL, Inc., Burlington, MA, USA). The backward differentiation formula (BDF) as an implicit time-dependent solver with the maximum order of accuracy of five and the time step of 2 s was utilized to solve Eq. (1).

## Results

### Analysis of mesh independence

**Figure 4:**
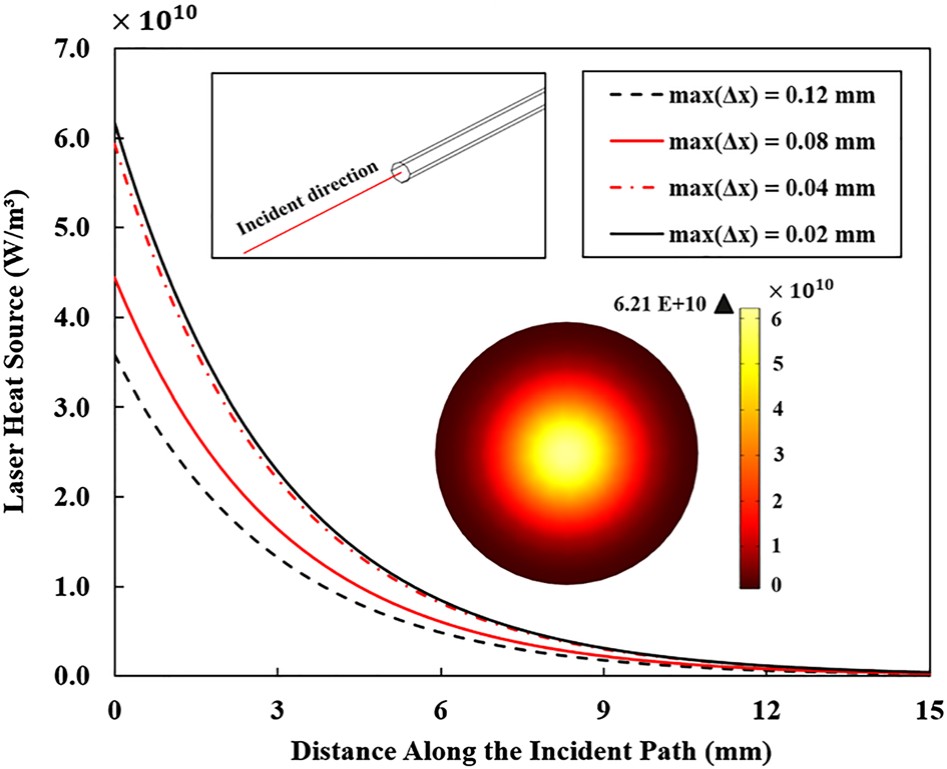
Analysis of the mesh independence based on the distribution of laser heat source along the direction of the incident laser beam at the laser power of 3 W, and based on the tissue optical properties presented in^16^.

Mesh independence is an important part of computational modeling to ensure that the results are not influenced by the element size and to determine the optimal mesh to reduce the computational cost. To ensure accurate capture of laser heat deposition, the mesh size must be sufficiently fine. In order to determine the ideal grid size, we examined maximum element sizes ranging from coarse to fine within the laser-heated domain (i.e. Δx = 0.12 mm, Δx = 0.08 mm, Δx = 0.04 mm, and Δx = 0.02 mm). The optimum mesh size was established once the heat source distribution converged (Fig. 4). According to Fig. 4, the laser heat source can be correctly captured along the incident direction when the maximum element size of Δx = 0.04 mm is utilized, approximately equivalent to one-tenth the diameter of the laser fiber. For the laser heat domain, swept mesh type was used while for the remaining tissue, tetrahedral mesh was applied. The smoother heat distribution is achieved by using swept mesh along the incident path.

### Comparing simulation results with ex vivo experimental data

**Figure 5:**
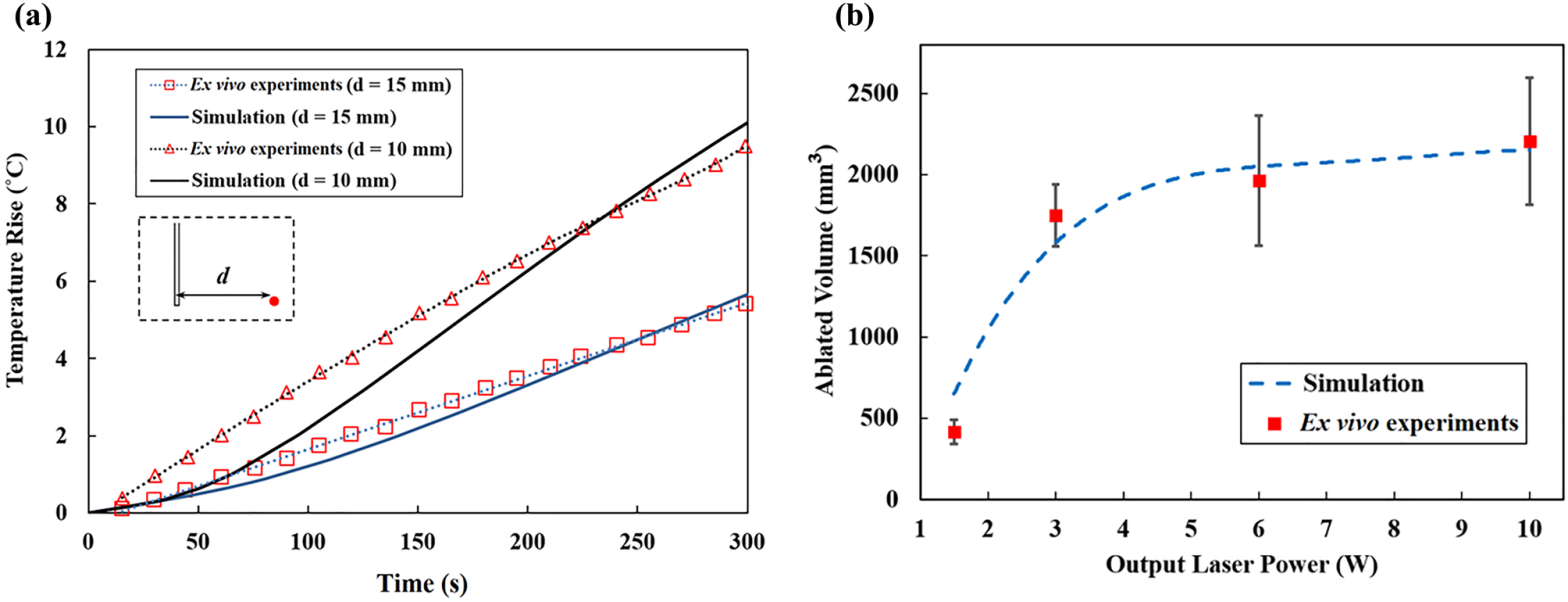
Comparison between the thermal results obtained from the present simulation and the results from ex vivo tests conducted on the porcine pancreas by^16^; (**a**) the temperature rise at the radial distances of 10 mm and 15 mm from the laser tip at the laser power of 3 W, (**b**) the obtained ablated volume as a function of the output laser power, 1.5 to 10 W.

As shown in Fig. 5, the simulation results for the laser-pancreatic tissue interaction were compared to those previously reported for 40 porcine healthy pancreases underwent LITT ex vivo^16^. The simulation settings follow those of reference experiments^16^ in which a quartz optical fiber with a core diameter of 300 µm was used to emit an Nd:YAG laser beam (1064 nm). As in this section the present simulation mimics ex vivo conditions, the parameters of blood perfusion and metabolic heat rate were both set to zero. Figure 5a presents the temporal temperature distribution at lateral distances of 10 and 15 mm from the fiber core when a 3 W laser power was applied, and Fig. 5b displays the ablated volumes at output laser powers ranging from 1.5 to 10 W. The temperature distribution in the experiments^16^ was recorded by six fiber Bragg grating sensors at fixed distances from the laser fiber, each with a 1 cm grating length. The discrepancy of predicted temperature rise from experiment records is higher during the initial period of irradiation, but it reduces with time. In order to measure the ablated volumes in^16^, the organs of the animals were examined histologically right after LITT. The authors sliced the organs and identified the LITT-induced thermal lesions for analysis. The area of the lesion in each slice was determined using Nikon System Software Arkon (Nikon Instruments S.p.A., Calenzano Florence, Italy); and the calculated area was then multiplied by the thickness of the corresponding slice^16^. Based on our simulations and the ex vivo experiments in^16^, the gradient of the ablated volume decreases with increasing laser power until it approaches a plateau at a laser power of 8 W, similar to the trend observed by Saccomandi et al.^44^ and Wu et al.^45^. According to Fig. 5, the simulation-based results well correlate with the results of ex vivo tests, implying that the model implementation is correct and can be further assessed for in vivo preclinical evaluation as outlined in the following sections.

### Comparing simulation results with in vivo clinical study

**Figure 6:**
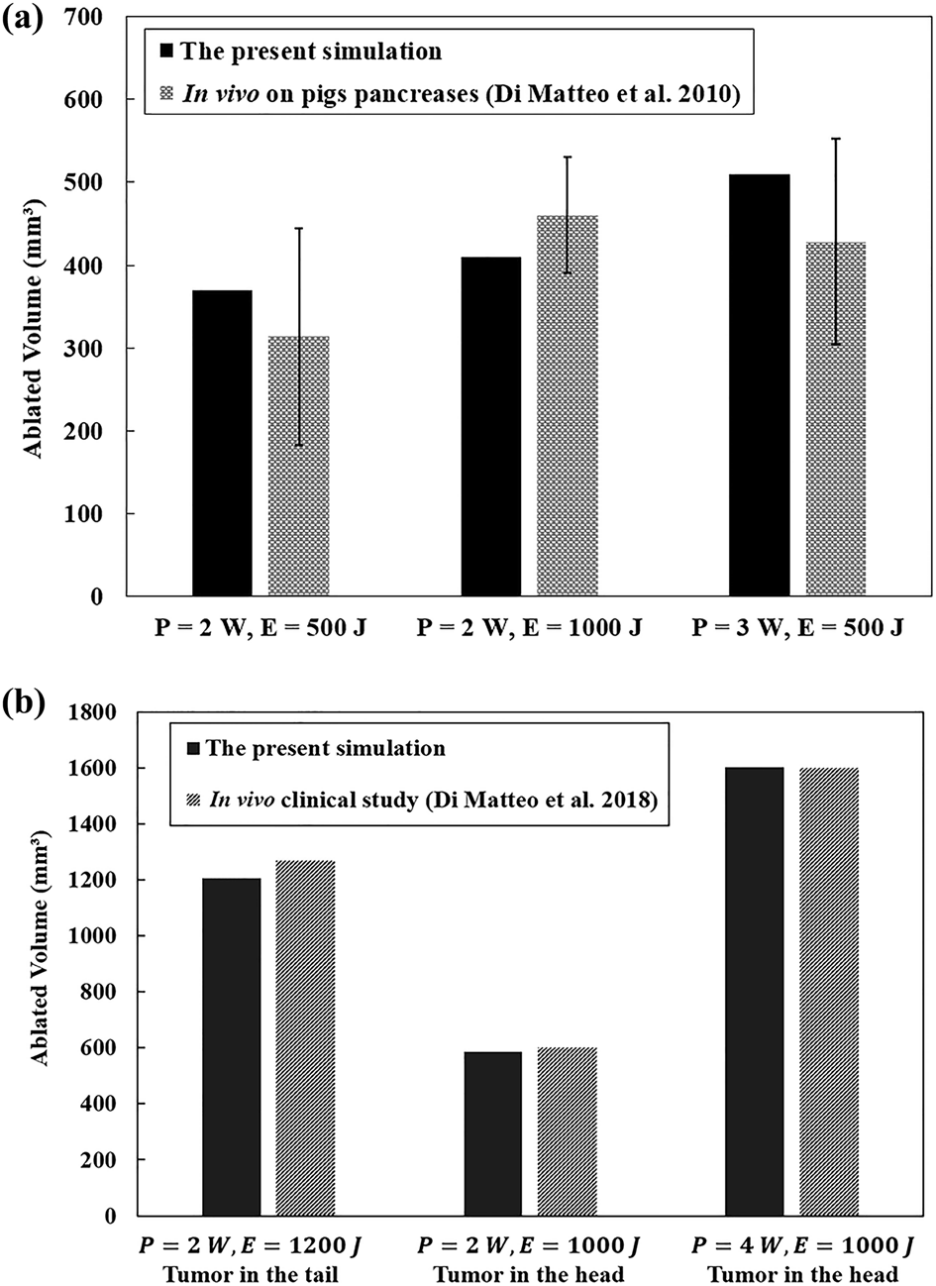
Comparison of ablated volumes predicted by the present simulation with those measured in (**a**) a pilot study on pigs^46^, (**b**) the clinical trial described in^6^.

To verify the model in vivo, the published data obtained from a pilot study of LITT on pigs are used with laser powers of 2 W at 500 J and 1000 J, and 3 W at 500 J (Fig. 6a). The target organ in^46^ is the healthy pancreas and the laser configuration and fiber diameter are the same as in the present work (300 µm, 1064 nm). Interventions were performed on the tail and body of the pancreas via a transgastric approach^46^. The simulation was remodeled to irradiate the healthy pancreases, the same as the condition of the experiments. According to Fig. 6a, a good agreement can be found between the simulation results and the in vivo experiments, with the discrepancy that can be attributed to the unknown placement of laser fiber. In Fig. 6a, it can be seen that, for the same power, as the laser energy (which is attributed to the irradiation time) increases, the ablation volume also increases. For example, when the power is held constant at 2 W, increasing the laser energy from 500 to 1000 J results in a larger ablation volume. When the energy level is held constant, increasing the power results in a larger ablation volume. For instance, when the energy is held constant at 500 J, increasing the power from 2 to 3 W results in a larger ablation volume. There is a clinical study^6^ that used 300 µm bare fiber with 1064 nm wavelength Nd:YAG laser light for the ablation of unresectable pancreatic adenocarcinomas at 2 W (800 J, 1000 J, and 1200 J), 3 W (800 J, 1000 J, and 1200 J), and 4 W (800 and 1000 J). A comparison is also made between the ablated volumes obtained from the current simulation and those obtained from a prior clinical trial^6^ for the treatment of PDAC tumors, as shown in Fig. 6b. The ground truth data for this comparison are those related to the measurement of ablated volume 1 month from the intervention in order to compensate for possible imaging artifacts during the early detection of the laser-induced lesion. Besides, we excluded the data in which the tumor underwent radiotherapy since it may alter the mechanical properties, such as stiffness of the tumor and consequently lead to a different pattern of the ablated zone. Based on the comparison of the data in Fig. 6b, the resultant ablated tumor volume in the pancreatic head is smaller than the one in the pancreatic tail although the laser was emitted at relatively close energies, 1000–1200 J. Most likely, the difference in the extent of ablated zones emanates from the high heat sink effect caused by blood perfusion in the duodenum and circulation through the inferior vena cava, superior mesenteric vein, and artery in the vicinity of the pancreatic head. This trend is also affirmed in a previous study employing the irreversible electroporation method for ablation of pancreatic tissue in vivo^47^. According to Fig. 6b, our prediction well matches the clinical data, hence, the present work may be useful for preclinical planning of LITT of the PDAC tumors removal.

### Cross-sectional temperature distribution

**Figure 7:**
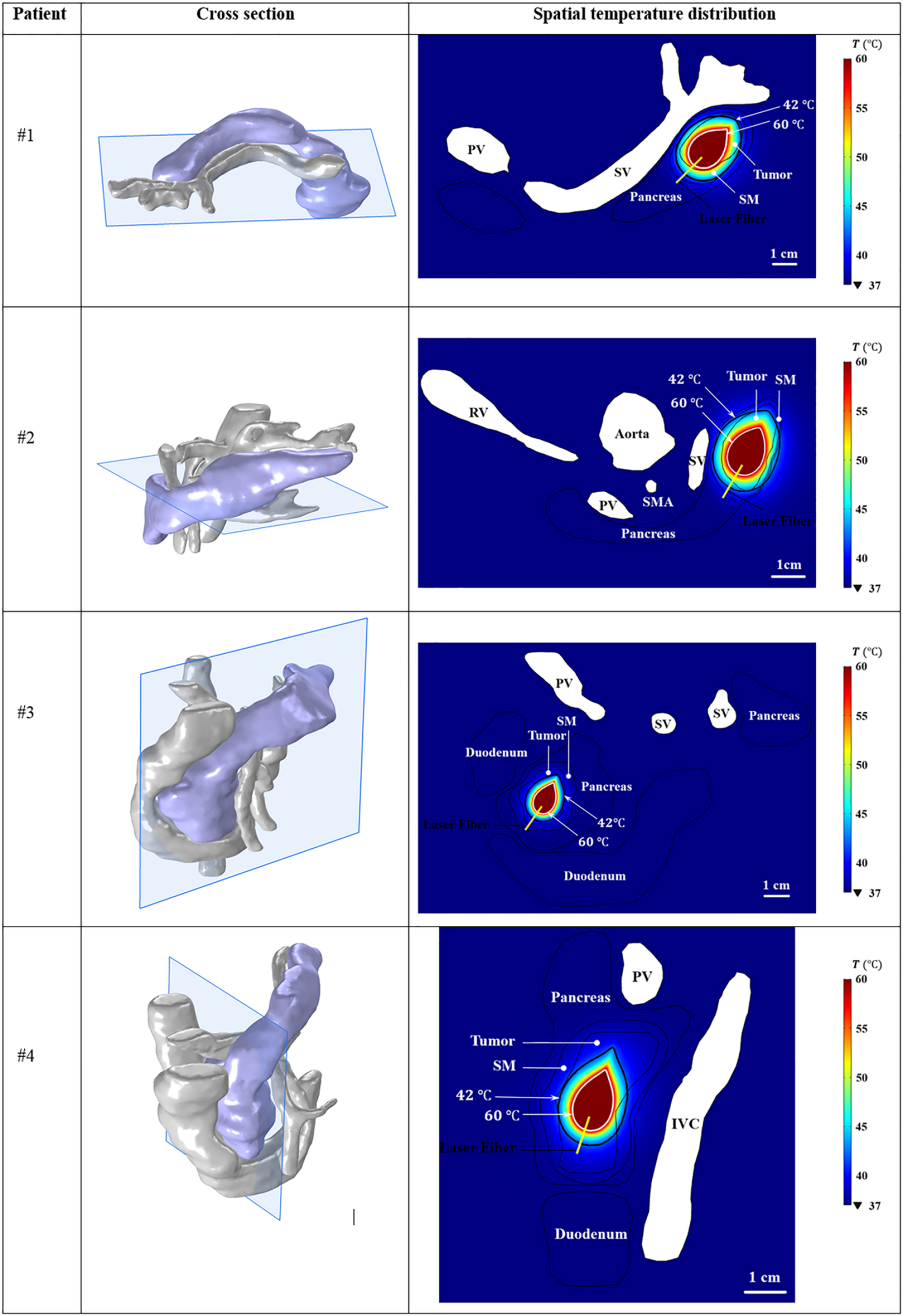
Spatial temperature distribution on the cross-sectional surface obtained from the output laser power of 2 W and laser energy of 1000 J; the black and white contours correspond to the temperature of 42 °C and 60 °C, respectively. The images were extracted using COMSOL Multiphysics software version 5.5 (COMSOL, Inc., Burlington, MA, USA, https://www.comsol.com/). *SM* safety margin, *SV* splenic vein, *PV* portal vein, *RV* renal vein, *SMA* superior mesenteric artery, *IVC* inferior vena cava.

The temperature distribution obtained from the applied laser power of 2 W and energy of 1000 J on the cross-sectional surface passing through the axis of laser fiber is illustrated in Fig. 7. Two contours are specified based on the temperature thresholds of 42 °C and 60 °C to define the regions under mild and ablative hyperthermia, respectively. The thermal ablation region, defined as the area where the fraction of thermal necrosis (Ω) is equal to 1 and calculated using Eqs. (6) and (7), is found to be similar in size to the region exposed to a temperature threshold of 60 °C. Based on clinical data^48^, it has been observed that exposure of living tissue to temperatures above 50 °C results in exponential decreases in the duration of exposure required to cause thermal damage. The effect of temperature on thermal ablation at such a level is more dominant than that of time, which is why, at 60 °C, thermal ablation occurs instantaneously^7,49,50^. A temperature threshold of 42 °C is also indicated in the temperature distribution to highlight the sublethal damage that should not be applied to vessels. The ablative hyperthermia leads to irreversible thermal damage of tissue while mild hyperthermia boosts the blood flow, which makes the tissue more susceptible to subsequent radiotherapy and/or chemotherapy. In Fig. 7, the ablated region has occurred within the tumor, while the mild hyperthermia region covers a part of the safety margin as well. The ablated area is $140 m{m}^{2}$, $143 m{m}^{2}$, $91 m{m}^{2}$, and $95 m{m}^{2}$ for patients #1 to #4, respectively. The lower thermal lesion sizes in patients #3 and #4, whose tumors are situated in the head of the pancreas, can be related to the higher heat dissipation due to the presence of inferior vena cava and duodenum, which is a highly-perfused organ.

### Optimization data for the LITT

The results of the optimization of the output laser power and exposure time are presented in Table 4 for patients #1 to #4, respectively, in terms of percentage of the ablated volume with respect to the total volume of tumor and safety margin (as a desirable outcome), as well as the maximum temperature of the surrounding organ at risk (as a constraint). For the sake of optimization, different percentages were tested, from 55 to 80%, and 55% was selected for our criteria since higher percentages showed high risk of thermal damage to the adjacent healthy tissues. Indeed, the safety margin volume is 0.85, 0.9, 1.6, and 1.2 times the tumor volume, for patients #1 to #4, accordingly; therefore, reaching 55% of the ablated volume within the safety margin zone guarantees complete tumor treatment.

**Table 4:**
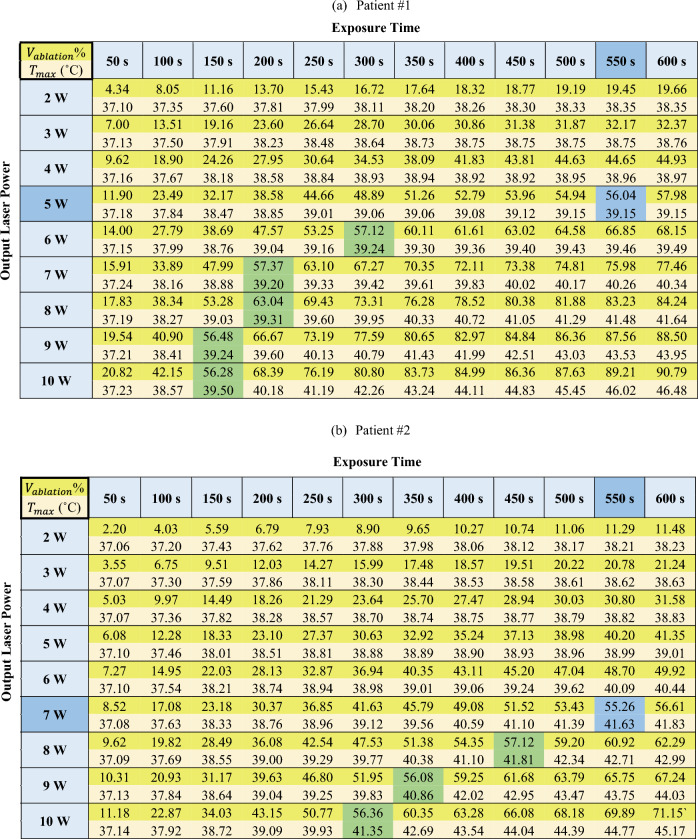

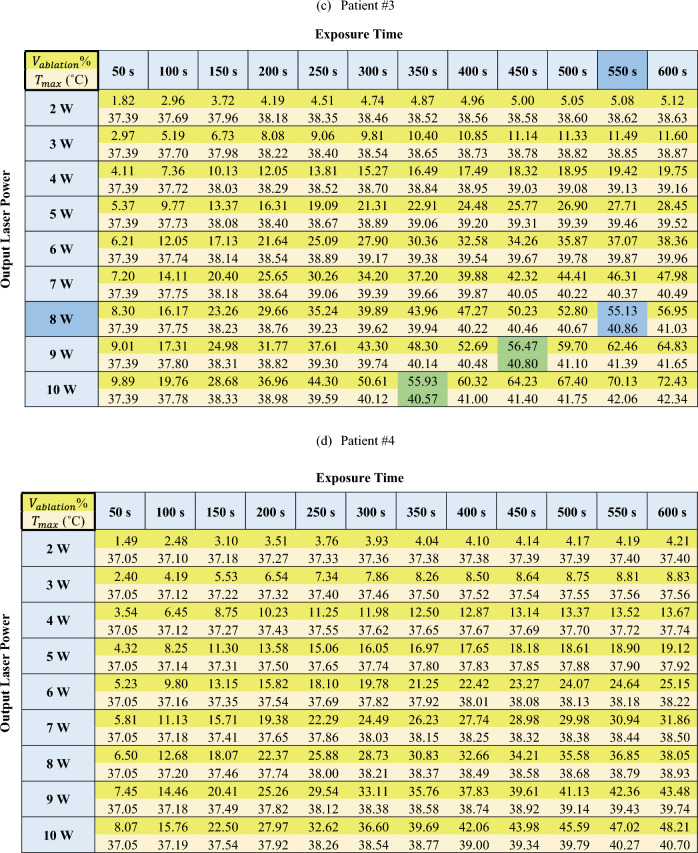
Percentage of ablated volume and maximum temperature of the organ at risk at various output laser powers and exposure times for (a) patient #1, (b) patient #2, (c) patient #3, and (d) patient #4.

Dark yellow: percentage of the ablated volume with respect to the total volume of tumor and safety margin; Light yellow: maximum temperature of the surrounding organ at risk; Green: values of ablated volume close to > 55%; Blue: preferred laser setting combination, based on the values of ablated volume close to > 55% and combined with the minimum output laser power.

Among the different combinations of $P$ and $t$ that provided the values close to > 55% (indicated by the green shade of color in Table 4), the combination with the minimum output laser power was preferred (indicated by the blue shade of color in Table 4).

Indeed, it is possible that two combinations of power and time result in the same ablation volume. For example, the highlighted cells in Table 4 results in the same ablation volume for patient #2: 7 W at 3850 J, 8 W at 3600 J, 9 W at 3150 J and 10 W at 3000 J provide approximately the same ablation volume.

Although higher power requires less energy or shorter period of irradiation to induce the same ablation volume, using high laser power can cause undesired effects, such as an increase in carbonization volume and degradation of the laser tip^11,32^. For this reason, the procedure should be performed at the lowest suggested power to guarantee the safety of the treatment and, at the same time, the therapy efficacy.

Hence, according to the results, the optimized output laser power and energy ($E=P\cdot t)$ for the treatment of patients #1, #2, and #3 are 5 W and 2750 J, 7 W and 3850 J, 8 W and 4400 J, respectively. Since the cancer was spread in a large portion of the pancreatic head in patient #4, the optimal laser setting was not achieved according to the prediction performed within the operating range. Multi-fiber laser ablation or pulled back technique with a single fiber, in which multiple ablations are executed at specified increments in order to cover the whole tumor^45,51,52^, can be explored as alternatives for the ablation of large tumors such as the one in patient #4.

**Figure 8:**
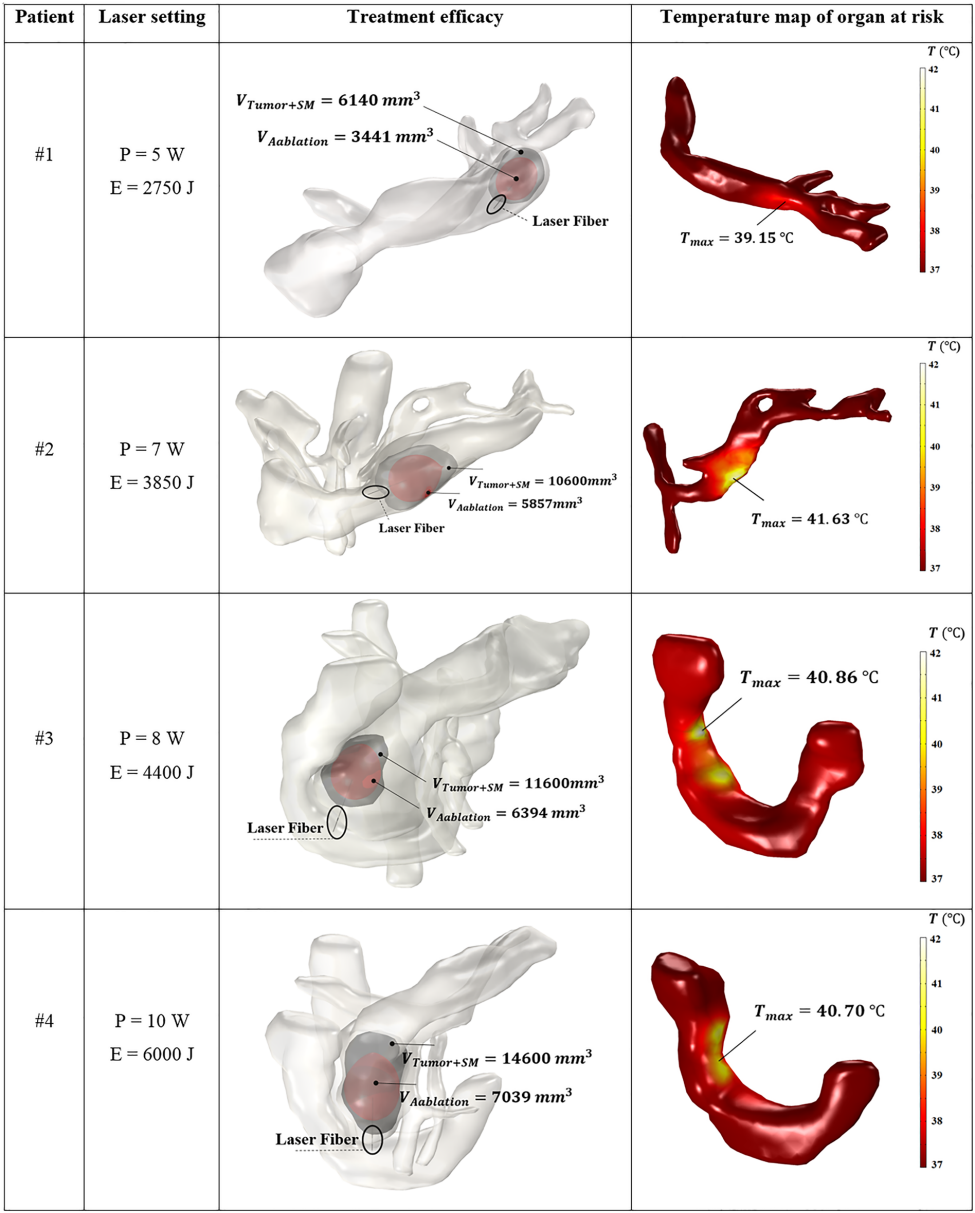
Attained ablated volume (red surface) within the safety margin zone (gray surface) and the temperature distribution of the organ at risk of thermal injury in patients #1 to #4. *SM* safety margin.

As shown in Fig. 8, the isothermal contour of the ablated volume at the obtained optimized laser dose is presented along with the temperature distribution on the surface of the organ most susceptible to thermal damage. According to the laser fiber placement in Fig. 8, the transduodenal approach was used for the treatment. A teardrop-shaped coagulation zone is visible in Fig. 8, as observed in previous experiments^11,45,53^. The organ at risk during the laser irradiation is the splenic vein in patients #1 and #2 and the duodenum in patients #3 and #4. A part of the vessel and the duodenum wall that is close to the lateral and lateral-posterior sides of the laser heating zone experience a higher temperature rise. The maximum temperature of the organ at risk was captured to be about 39 °C (5 W) for patient #1, 42 °C (7 W) for patient #2, 41 °C (8 W) for patient #3, and 41 °C (10 W) for patient #4 at the end of irradiation time ($\sim$ 600 s).

**Figure 9:**
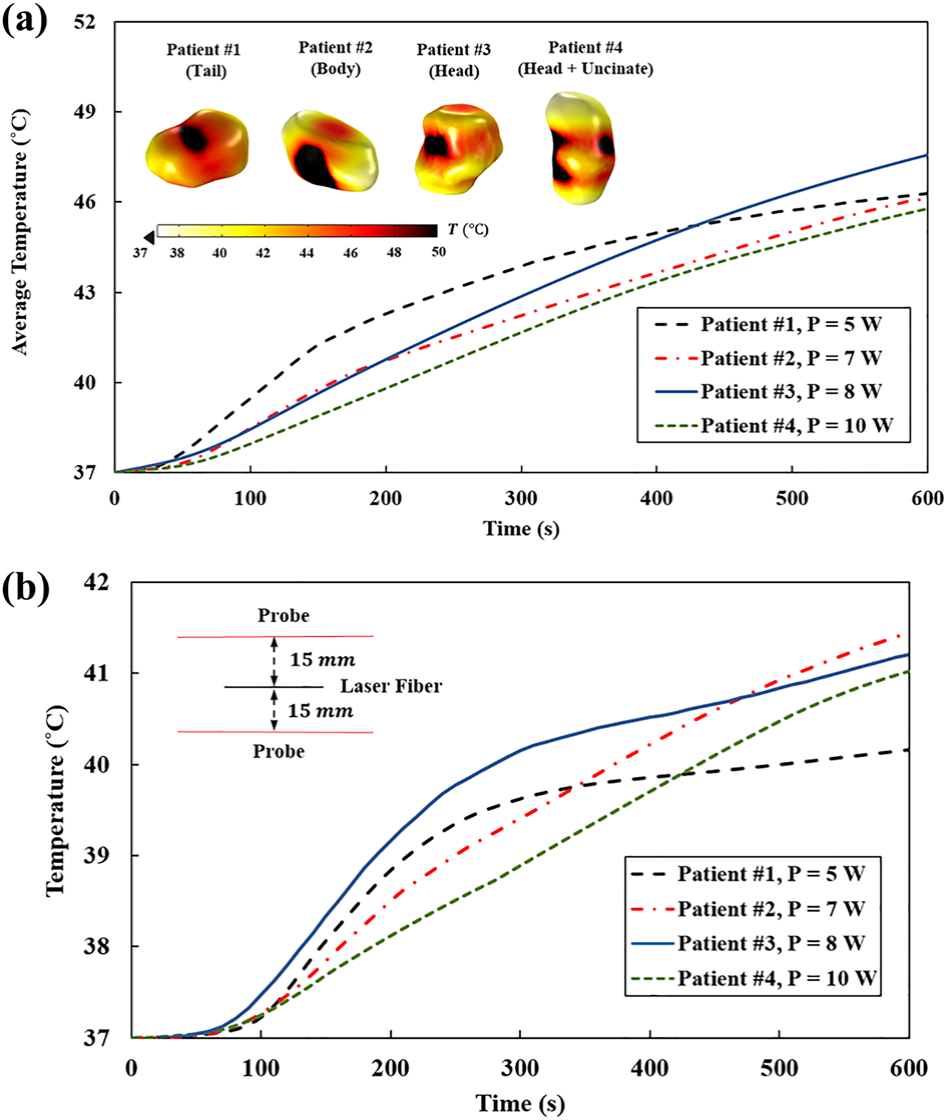
(**a**) Average temperature of the safety margin surface and (**b**) maximum temperature recorded by the probes placed at an interval of 15 mm from the laser fiber, as a function of time.

The spatio-temporal temperature patterns of the safety margin at the optimized laser powers are shown in Fig. 9a. The graph shows the average temperature of the safety margin surface over time and the 3D maps show the temperature distributions at the end of exposure time. The regions with the temperature range of 40–42 °C (mild hyperthermia) do not experience thermal cytotoxicity, however, the chemo/radio-sensitization of tumor cells may be promoted as the oxygenation increases by the stimulation of blood flow. The regions within the temperature range of 42–45 °C (moderate hyperthermia) undergo DNA damage and apoptosis, triggering the anti-tumor immune response. In regions with a temperature above 50 °C (thermal ablation), protein denaturation and rapid coagulation necrosis occur; meanwhile, the vascular shutdown accentuates the intratumoral acidification which has a synergistic effect on tumor cell death^7,40,54^. It should be noted that hyperthermia at a high-temperature range can alleviate the extracellular matrix elasticity and relieve the interstitial fluid pressure which facilitates the formation of the vasculature network and regrowth of the tumor, unless the entire tumor is completely ablated^55^.

To ensure the safety of using the optimized laser doses, the maximum temperature of tissue was recorded by two probes defined in the simulation model and placed at a lateral distance of 5 mm away from the safety margin or 15 mm from the laser fiber (Fig. 9b). Results show that the maximum temperature has not exceeded 42 °C, which is the threshold for thermal damage. There is a sharp temperature gradient from 37 °C to 40 °C; conversely, when the temperature surpasses 40 °C, the tissue blood flow increases as well as its heat sink effect, thus smoother temperature rise is observed.

## Discussion and conclusion

On the basis of patient-specific anatomical models, in silico predictions were made concerning the thermal response of exposed tissues to laser irradiation during the interstitial thermotherapy of PDAC tumors. The optimization of laser dosimetry was carried out to obtain a complete thermal ablation of tumors situated at different locations of the pancreas, including the tail, the body, and the head while preserving the neighboring healthy organs. Various optical-thermal phenomena such as scattering and absorption of light, energy changes due to the tissue water evaporation, heat sink effects due to the blood perfusion in the capillaries, and blood circulation through the major vessels were taken into consideration in the implemented bioheat model for the laser-tissue interaction. By comparing the size of the laser-induced coagulation zones, it was revealed that the pancreatic head is affected by more heat loss, which requires a higher laser intensity to achieve the same level of tumor ablation. At the optimal laser power and energy per patient case, adjacent vital organs did not experience temperatures exceeding 42 °C. Besides, at an interval of 15 mm along the lateral sides of the laser fiber, no temperature higher than 42 °C was recorded. The other objective of this study was to produce thermal damage to a margin around the tumor, i.e. safety margin, in order to eliminate the remnant tumor cells. Although the thorough thermal ablation was not achieved within the safety margin by applying the optimized laser settings, the induced temperature values were high enough to possibly cause DNA damage to tumor cells while impeding DNA repair, stimulating anti-tumor immune responses, and sensitizing the tumor cells to the following chemo-/radio- therapy^55^.

The predicted results are in good quantitative and trend-wise agreement with the previous studies of ex vivo porcine pancreas and in vivo human PDAC tumors. However, most of the ablation sessions in clinical trials were performed at a relatively low laser energy level and there are no clinical treatment data available for the Nd:YAG laser (1064 nm) with the output powers and energies greater than 4W/1200 J. Therefore, more clinical data are necessary to verify the predicted results at high laser powers. The other important factor that highly influences the efficacy of thermotherapy is the blood perfusion rate. In response to hyperthermia, healthy tissue shows a significantly different pattern of blood flow, up to tenfold higher at 42–48 °C, than tumorous tissue^41^. In addition, the pattern of blood perfusion in the healthy and tumor tissue itself can even be different, depending on the type of the tissue (e.g. skin, muscle, fat, etc.). During tumor growth, its demand for nutrients from the host vasculature increases; however, at a specific stage, when the tumor's interstitial pressure surpasses the arterioles' vascular pressure, local vascular stasis occurs and blood cannot be supplied to the central areas of the tumor, so these areas become hypoxic and necrotic. A significant correlation has been found between hypoxia extent and the distribution of blood perfusion, depending on the tumor stage, size, and type^56^. Accordingly, the tumor blood flow is a critical uncertainty in the estimation of laser-induced thermal effects, and it is better to measure this parameter for each tumor individually before running the computer-based pretreatment planning. Last but not the least, the exact temperature threshold for defining the border of hyperthermic lesions is unclear, as different values have been reported in the literature between 50 and 60 °C^7,25,31,57,58^. In spite of the uncertainties described above, the present study used the consensus set of parameters.

In conclusion, the present paper, by examining the therapeutic outcome of laser thermotherapy, as measured by the size of the ablation volumes and at a wide range of output laser powers, can provide useful information for physicians to adjust the appropriate laser parameters. The methods and the results of this study represent the first solid bases for the development of a preplanning LITT platform that involves the individual patient anatomy in the decision-making process of the selection of the procedural settings. This would imply multiple potential clinical benefits. Indeed, the pre-procedural definition of patient-tailored settings could guarantee customized and, thus, more effective ablation volumes, thanks to the prediction of the ablative effect on the whole tumor, including safety margins. Moreover, the validation of this mathematical model would significantly contribute in reducing procedure-related complications thanks to the heat sink effect prediction on surrounding tissues. These advantages may thus, lead, in the near future, to a wider and safer application of LITT even to difficult clinical cases (i.e. excessive tumor proximity to major vessels or duodenum), giving a chance to patients currently excluded from ablative treatments. In addition, although to be investigated, it is likely that such a patient-tailored model could bring advantages even in terms of long-term prognosis (overall survival and progression-free survival) as compared to current palliative treatments.

In future, a dedicated clinical study will be outlined, with the aim to validate the developed patient-specific model in patients undergoing LITT with the procedural setting predicted by the model.

## Data Availability

All data used for this study are available from the author upon request. For requesting data produced in this study, please contact the corresponding author.
